# Supplementary figures and images for: Enhancement of loop-mediated isothermal amplification (LAMP) with guanidine hydrochloride for the detection of Streptococcus equi subspecies equi (Strangles)
Source: PeerJ. 2024 Oct 8;12:e17955. doi: 10.7717/peerj.17955 (PMC11484460; doi:10.7717/peerj.17955)

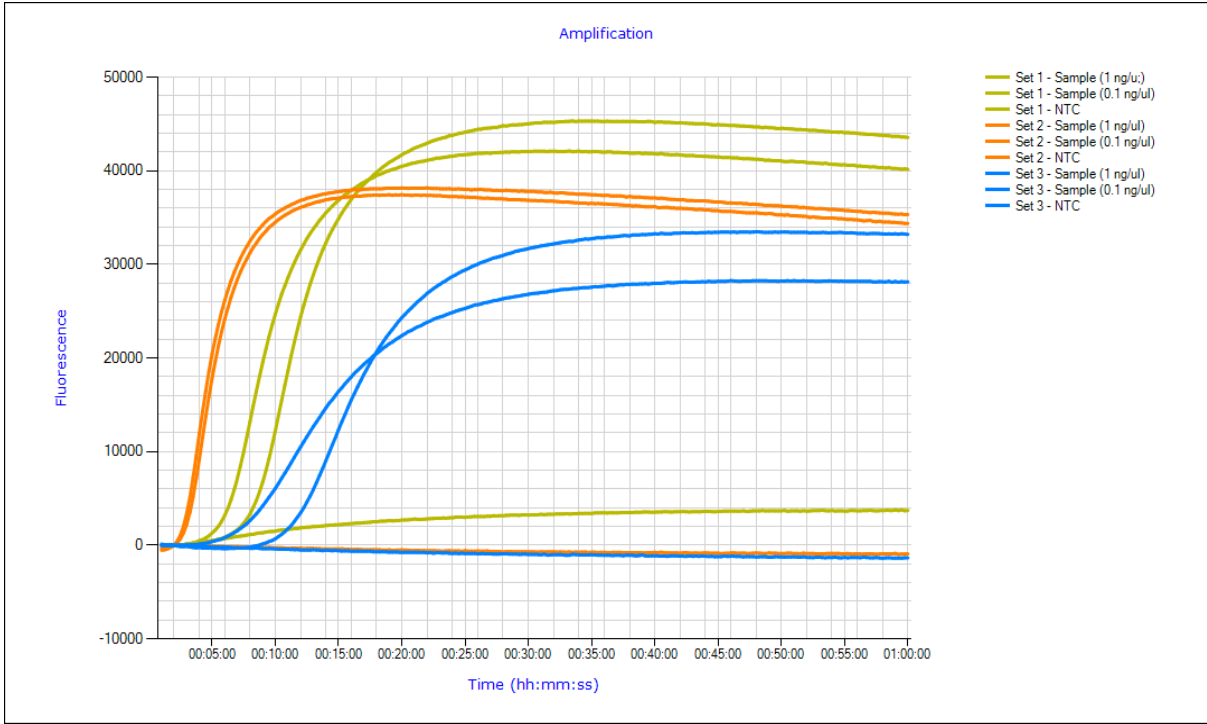

Supplement: Supplemental Information 1 — Each primer set was evaluated using 1 ng/µl and 0.1 ng/µl of the synthetic positive control, and a no template control (NTC). Primer set 1 is represented in gold, primer set 2 is represented in orange, and primer set 3 is represented in blue. NTCs for each primer set are denoted in respective colours. [file peerj-12-17955-s001.pdf]

**C**

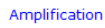

Supplement: Supplemental Information 2 — Specificity panel tested against the Str-LAMP assay of bacteria other than Streptococcus species, across three separate assays (Fig S3a-c). Each assay was performed with the Str-LAMP synthetic positive control (+ve con) and a no template control (NTC). [file peerj-12-17955-s002.pdf]
