# Supplementary material for: Enhancement of loop-mediated isothermal amplification (LAMP) with guanidine hydrochloride for the detection of Streptococcus equi subspecies equi (Strangles)
Source: PeerJ. 2024 Oct 8;12:e17955. doi: 10.7717/peerj.17955 (PMC11484460; doi:10.7717/peerj.17955)
Supplement: Supplemental Information 3 — Pairwise alignment of the closely related pathogen Streptococcus equi subspecies zooepidemicus (GenBank acc no: JX014303.1 ) M gene (SzM) against Streptococcus equi subspecies equi (GenBank acc no: U73162 ) M gene (SeM). The Str-LAMP assay primers are highlighted throughout, outer primers (F3 and B3) highlighted in yellow, the forward inner primers (FIP) and backwards inner primers (BIP) are highlighted in yellow, and the loop backwards primer (LB) is highlighted in green. Sequence consensuses are highlighted in red. Alignment was performed using Benchling online software (Benchling, San Fransico, United States), and was edited in Microsoft Word (Microsoft Corporation, Washington, United States). [file peerj-12-17955-s003.pdf]

```
                411                                                                          492
U73162.1 ...    GTATTAGTTGCAACAAGTGTGTTGGGAGGGACAACTGTAAAAGCGAACTCTGAGGTTAGTCGTACGGCGACTCCAAGATTAT
>JX014303...    GTGTA------TGCTCGTTATTTACGTGGTCTTA---------------ATGATGATGTTCAAAAGATTTATGACAGGTTGA

..........................................................................................

                493                                                                          574
U73162.1 ...    CGCGTGATTTAAAAAATAGATTAAGCGATATAGCCATAAGTGGAGATGCCTCATCAGCCCAAAAAGTTCGAAATCTTCTAAA
>JX014303...    CAGGCGATGATGTCAGAGAATTATTAGGAAAAGAAATAGAGAAAAGTACAGGCCTAGAGCAAACAATTGGGGAATTAACTTT

..........................................................................................

                575                                                                          656
U73162.1 ...    GGCGCCTCTGTTGGGGATTTACAGGCATTATTGAGAGGTCTTGATTCAGCAAGGGCTGCGTATGGTAGAGATGATGATTATT
>JX014303...    -AACAAATAGTAGTCTTCGTGA-TAAATTAGAAGATCTAAAAGAGACTTTAGCTACAACTGCTCTTGCGATTCAAGAGGAAA

..........................................................................................
```
