# Supplementary material for: Enhancement of loop-mediated isothermal amplification (LAMP) with guanidine hydrochloride for the detection of Streptococcus equi subspecies equi (Strangles)
Source: PeerJ. 2024 Oct 8;12:e17955. doi: 10.7717/peerj.17955 (PMC11484460; doi:10.7717/peerj.17955)
Supplement: Supplemental Information 4 [file peerj-12-17955-s004.docx]

**Supplemental Table S1**

**Table S1:** Limit of detection (LOD) for primer concentration 3 for the *Streptococcus equi* subspecies *equi* loop-mediated isothermal amplification (Str-LAMP) assay.

| DNA concentration  (ng/µl) | Av Tp^1^  (mm:ss) | SD^2^ (mm:ss) |
| --- | --- | --- |
| 1x10^0^ | 05:02 | 00:07 |
| 1x10^-1^ | 06:25 | 00:05 |
| 1x10^-2^ | 08:49 | 00:18 |
| 1x10^-3^ | 10:38 | 00:15 |
| 1x10^-4^ | 12:33 | 00:57 |
| 1x10^-5^ | 13:06 | 00:24 |
| 1x10^-6^ | No amplification |  |
| 1x10^-7^ | No amplification |  |
| 1x10^-8^ | No amplification |  |
| 1x10^-9^ | No amplification |  |

^1^ Average time to positive (Tp) across 3 technical replicates

^2^ Standard deviation (SD) of Tp across 3 technical replicates
