# Supplementary material for: Enhancement of loop-mediated isothermal amplification (LAMP) with guanidine hydrochloride for the detection of Streptococcus equi subspecies equi (Strangles)
Source: PeerJ. 2024 Oct 8;12:e17955. doi: 10.7717/peerj.17955 (PMC11484460; doi:10.7717/peerj.17955)
Supplement: Supplemental Information 5 [file peerj-12-17955-s005.docx]

**Supplemental Table S2**

**Table S2:** Full results for *Streptococcus equi* subspecies *equi* and *S. zooepidemicus* clinical isolates.

| Sample ID | Av Tp (mm:ss) | Result | Correct classification? |
| --- | --- | --- | --- |
| SEE 1 | 13:44 | POS | Y |
| SEE 2 | 13:34 | POS | Y |
| SEE 3 | 13:53 | POS | Y |
| SEE 4 | 14:25 | POS | Y |
| SEE 5 | 26:07 | NEG | N |
| SEE 6 | 22:14 | NEG | N |
| SEE 7 | 10:17 | POS | Y |
| SEE 8 | 10:19 | POS | Y |
| SEE 9 | 10:17 | POS | Y |
| SEE 10 | 11:11 | POS | Y |
| SEE 11 | 11:43 | POS | Y |
| SEE 12 | 11:31 | POS | Y |
| SEE 13 | 11:03 | POS | Y |
| SEE 14 | 11:27 | POS | Y |
| SEE 15 | 11:08 | POS | Y |
| SEE 16 | 14:25 | POS | Y |
| SEE 17 | 16:11 | POS | Y |
| SEE 18 | 13:30 | POS | Y |
| SEE 19 | 14:09 | POS | Y |
| SEE 20 | 13:27 | POS | Y |
| SEE 21 | 12:51 | POS | Y |
| SEE 22 | 13:36 | POS | Y |
| SEE 23 | 12:52 | POS | Y |
| SEE 24 | 09:51 | POS | Y |
| SEE 25 | 09:47 | POS | Y |
| SEE 26 | 10:04 | POS | Y |
| SEE 27 | 10:03 | POS | Y |
| SEE 28 | 09:23 | POS | Y |
| SEE 29 | 09:07 | POS | Y |
| SEE 30 | 09:07 | POS | Y |
| SEE 31 | 09:31 | POS | Y |
| SEE 32 | 14:35 | POS | Y |
| SEE 33 | 14:24 | POS | Y |
| SEE 34 | 14:40 | POS | Y |
| SEE 35 | NA | NEG | N |
| SEE 36 | 14:15 | POS | Y |
| SEE 37 | 13:39 | POS | Y |
| SEE 38 | 14:52 | POS | Y |
| SEE 39 | 15:24 | POS | Y |
| SEE 40 | 11:46 | POS | Y |
| SEE 41 | NA | NEG | N |
| SEE 42 | 13:56 | POS | Y |
| SEE 43 | 14:36 | POS | Y |
| SEE 44 | NA | NEG | N |
| SEE 45 | 28:54 | NEG | N |
| SEE 46 | 12:49 | POS | Y |
| SEE 47 | 13:40 | POS | Y |
| SEE 48 | 14:26 | POS | Y |
| SEE 49 | 15:07 | POS | Y |
| SEE 50 | 18:45 | POS | Y |
| SEE 51 | 19:27 | POS | Y |
| SEE 52 | 15:12 | POS | Y |
| SEE 53 | 18:55 | POS | Y |
| SEE 54 | 13:01 | POS | Y |
| SEE 55 | 12:05 | POS | Y |
| SEE 56 | 11:07 | POS | Y |
| SEE 57 | 11:13 | POS | Y |
| SEE 58 | 13:26 | POS | Y |
| SEE 59 | 12:33 | POS | Y |
| SEE 60 | 11:51 | POS | Y |
| SEE 61 | 11:49 | POS | Y |
| SEE 62 | 11:36 | POS | Y |
| SEE 63 | 12:02 | POS | Y |
| SEE 64 | 13:03 | POS | Y |
| SEE 65 | 12:48 | POS | Y |
| SEE 66 | 11:51 | POS | Y |
| SEE 67 | 11:38 | POS | Y |
| SEE 68 | 13:50 | POS | Y |
| SEE 69 | 13:45 | POS | Y |
| SEE 70 | 16:14 | POS | Y |
| SEE 71 | 14:49 | POS | Y |
| SEE 72 | 13:22 | POS | Y |
| SEE 73 | 12:18 | POS | Y |
| SEE 74 | 21:03 | NEG | N |
| SEE 75 | 14:43 | POS | Y |
| SEE 76 | 15:22 | POS | Y |
| SEE 77 | 16:06 | POS | Y |
| SEE 78 | 13:29 | POS | Y |
| SEE 79 | 18:39 | POS | Y |
| SEE 80 | 11:22 | POS | Y |
| SEE 81 | 11:22 | POS | Y |
| SEE 82 | 14:19 | POS | Y |
| SEE 83 | 14:22 | POS | Y |
| SEE 84 | 13:21 | POS | Y |
| SEE 85 | 13:23 | POS | Y |
| SEE 86 | 20:03 | NEG | N |
| SEE 87 | 18:30 | POS | Y |
| SEE 88 | 19:04 | POS | Y |
| SEE 89 | 15:51 | POS | Y |
| SEE 90 | 14:37 | POS | Y |
| SEE 91 | 13:23 | POS | Y |
| SEE 92 | 11:24 | POS | Y |
| SEE 93 | 11:34 | POS | Y |
| SEE 94 | 13:20 | POS | Y |
| SEE 95 | 13:26 | POS | Y |
| SEE 96 | 13:26 | POS | Y |
| SEE 97 | 13:31 | POS | Y |
| SEE 98 | 13:24 | POS | Y |
| SEE 99 | 13:16 | POS | Y |
| SEE 100 | 19:30 | POS | Y |
| SEE 101 | 23:34 | NEG | N |
| SEE 102 | 13:06 | POS | Y |
| SEE 103 | 12:50 | POS | Y |
| SEZ 1 | NA | NEG | Y |
| SEZ 2 | NA | NEG | Y |
| SEZ 3 | NA | NEG | Y |
| SEZ 4 | NA | NEG | Y |
| SEZ 5 | NA | NEG | Y |
| SEZ 6 | 24:34 | NEG | Y |
| SEZ 7 | NA | NEG | Y |
| SEZ 8 | NA | NEG | Y |
| SEZ 9 | NA | NEG | Y |
| SEZ 10 | 17:33 | POS | N |
| SEZ 11 | NA | NEG | Y |
| SEZ 12 | NA | NEG | Y |
| SEZ 13 | 19:35 | POS | N |
| SEZ 14 | NA | NEG | Y |
| SEZ 15 | NA | NEG | Y |
| SEZ 16 | 16:25 | POS | N |
| SEZ 17 | NA | NEG | Y |
| SEZ 18 | NA | NEG | Y |
| SEZ 19 | NA | NEG | Y |
| SEZ 20 | NA | NEG | Y |
| SEZ 21 | NA | NEG | Y |
| SEZ 22 | NA | NEG | Y |
| SEZ 23 | NA | NEG | Y |
| SEZ 24 | 25:14 | NEG | Y |
| SEZ 25 | 21:40 | NEG | Y |
| SEZ 26 | NA | NEG | Y |
| SEZ 27 | NA | NEG | Y |

SEE, *Streptococcus equi* subspecies *equi*; SEZ *Streptococcus equi* subspecies *zooepidemicus*; NA, no amplification
